# Proportional-Integral-Derivative (PID) Control of Secreted Factors for Blood Stem Cell Culture

**DOI:** 10.1371/journal.pone.0137392

**Published:** 2015-09-08

**Authors:** Julia Caldwell, Weijia Wang, Peter W. Zandstra

**Affiliations:** 1 Institute of Biomaterials and Biomedical Engineering, University of Toronto, Toronto, Ontario, Canada; 2 The Donnelly Centre, University of Toronto, Toronto, Ontario, Canada; 3 Department of Chemical Engineering and Applied Chemistry, University of Toronto, Toronto, Ontario, Canada; 4 McEwen Centre for Regenerative Medicine, University of Health Network, Toronto, Ontario, Canada; The Ohio State University, UNITED STATES

## Abstract

Clinical use of umbilical cord blood has typically been limited by the need to expand hematopoietic stem and progenitor cells (HSPC) ex vivo. This expansion is challenging due to the accumulation of secreted signaling factors in the culture that have a negative regulatory effect on HSPC output. Strategies for global regulation of these factors through dilution have been developed, but do not accommodate the dynamic nature or inherent variability of hematopoietic cell culture. We have developed a mathematical model to simulate the impact of feedback control on in vitro hematopoiesis, and used it to design a proportional-integral-derivative (PID) control algorithm. This algorithm was implemented with a fed-batch bioreactor to regulate the concentrations of secreted factors. Controlling the concentration of a key target factor, TGF-β1, through dilution limited the negative effect it had on HSPCs, and allowed global control of other similarly-produced inhibitory endogenous factors. The PID control algorithm effectively maintained the target soluble factor at the target concentration. We show that feedback controlled dilution is predicted to be a more cost effective dilution strategy compared to other open-loop strategies, and can enhance HSPC expansion in short term culture. This study demonstrates the utility of secreted factor process control strategies to optimize stem cell culture systems, and motivates the development of multi-analyte protein sensors to automate the manufacturing of cell therapies.

## Introduction

Hematopoietic stem cell (HSC) transplantation has been used for several decades to successfully treat various pathologies. Umbilical cord blood (UCB) derived HSCs offer several advantages over traditional bone marrow or mobilized peripheral blood derived HSCs, including robust long-term immune reconstitution and reduced incidence of graft-versus-host disease [1, 2]. However, lower cell numbers in UCB units have typically limited its use to pediatric patients [3]. A robust bioprocess to expand both HSCs for long-term engraftment and progenitor cells for short term hematopoietic recovery would allow this therapy to be widely used in adults. Such a bioprocess would also expand the application of HSCs into other areas, such as gene therapies for personalized, patient-specific treatments.

Expansion of HSCs ex vivo has proven difficult. Hematopoietic cell fate decisions are governed by a complex intercellular signaling network of endogenously produced and secreted soluble factors [4–6]. Cell-cell communication network analysis using gene expression data has shown that HSCs are responsive to many ligands secreted by other cell types, and therefore they are particularly sensitive to culture [6]. The soluble factors that accumulate in culture include many cytokines and chemokines that are primarily secreted by mature cell phenotypes and drive HSC cell fate decisions away from self-renewal and towards differentiation, resulting in an inhibitory feedback loop for the HSC [7, 8]. Strategies to expand HSCs have focused on small molecules to selectively expand HSCs [9–12] or open loop bioprocesses to remove secreted factors [13, 14]. The current best-in-class bioprocess combines these approaches, using a fed-batch dilution scheme in combination with a small molecule differentiation inhibitor, UM171 [15]. All these approaches do not address the complex dynamic nature of hematopoietic cell culture or the variability in input cell composition. Direct monitoring and control of the concentrations of these secreted factors would complement current expansion strategies and could increase the robustness and clinical efficacy of the bioprocess.

One process control strategy that has been used to improve HSC and progenitor cell (HSPC) expansion is to control physicochemical culture variables, such as temperature, pH and dissolved oxygen concentration [16–18]. Bioreactor systems have also incorporated feedback control of nutrients and metabolites to facilitate optimal cell growth [19]. Although the control of these variables is important, particularly for cell viability, these strategies do not address the parameters such as endogenous cytokine or morphogen concentrations that typically govern stem cell fate decisions. It is likely that current cell therapy bioprocesses can be improved by incorporating careful control of the concentrations of secreted factors that directly regulate cell proliferation and differentiation; herein we explore this hypothesis using HSPC cultures.

Using a very simple control algorithm, we previously demonstrated that real-time control dilution in response to the accumulation of secreted factors enhanced HSPC expansion, although with a high media volume requirement [20]. A more sophisticated feedback control strategy for secreted factor regulation could maintain the enhanced HSPC expansion while also optimizing media usage, creating a cost-effective and clinically relevant bioprocess for cord blood expansion.

Currently, no sophisticated feedback control systems for secreted factor regulation have been implemented in the production of cell therapy products. Furthermore, fundamental mathematical descriptions of cell dynamics and responses to changing environment have not been used to design and investigate such feedback controllers. Here, we describe the development of an empirical model of UCB-derived hematopoietic cell culture and its utility in designing a control strategy for efficient HSPC expansion. By combining an *in silico* optimized proportional-integral-derivative (PID) feedback controller with our fed-batch culture system, we experimentally validated predictions of improved total and CD34^+^ cell expansion over 12 days of culture. Furthermore, we show that this type of feedback control, complements other HSPC expansion strategies, including cultures supplemented with the recently discovered differentiation inhibitor UM729 [15]. This study demonstrates the feasibility of using feedback control as part of a next-generation bioprocess for patient-specific cell therapies.

## Materials and Methods

### Quantum Dot Microbead Preparation

Quantum dot (QD) barcoded microbeads were synthesized as previously reported [21]. Microbeads were conjugated with anti-latency associate peptide (LAP, a component of the TGF-β1 complex) capture antibody by incubating with the chemical cross linker 1-ethyl-3-(3-dimethylaminopropyl)carbodiimde (EDC) (1 mg/mL in MES buffer, 50 μM, pH 6.0, Sigma-Aldrich, St Louis, MO) for 30 minutes, followed by incubation with anti-LAP capture antibody (0.5 mg/mL in PBS, R&D Systems, Minneapolis, MN) overnight. Conjugated beads were resuspended at a final concentration of 2.0×10^7^ beads/mL.

### Latency Associated Peptide Detection Assay

As previously reported [20], each assay reaction contained 1 μL of QD microbeads, 1 μL of biotin labeled reporter antibody (25 μg/mL, R&D Systems), and 10 μL of a conditioned media sample. Reactions were carried out at 37°C for 1 hour. Streptavidin APC solution (1:1000) (BD Biosciences, San Jose, CA) was added to the samples and the reaction was carried out for 30 minutes. Microbeads were washed and analyzed using a FACSCanto flow cytometer (BD Biosciences). LAP microbeads were first identified and gated using the QD fluorescent signal, and the concentration of LAP was calculated using the APC signal.

### Secreted Factor Assays

Time course secreted factor analysis was performed on conditioned media samples using the Human Cytokine/Chemokine 41-plex Magnetic Bead Panel (Millipore, Billerica, MA), designed for the Luminex microsphere detection platform (Luminex Co., Austin, TX). TGF-β1 was analyzed in parallel using a TGF-β1 ELISA Kit (R&D Systems), according to the manufacturer’s directions.

### Umbilical Cord Blood Cell Processing and In Vitro Culture

Umbilical cord blood samples were collected from consenting donors at Mount Sinai Hospital (Toronto, ON). This procedure was approved by the Mount Sinai Hospital Research Ethics Board, and written consent was obtained. Mononuclear cells were obtained as described [22]. From this fraction, CD34^+^ cells were selected using *EasySep* (StemCell Technologies, Inc., Vancouver, BC) according to the manufacturer’s protocol. The selected CD34^+^ cells were plated at an initial density of 1.0×10^5^ cells/mL in serum-free IMDM media (GIBCO, Rockville, MD) with 20% BIT serum substitute (StemCell Technologies) and 1% Glutamax (GIBCO). Media was supplemented with 100 ng/mL SCF, 100 ng/mL Flt3L, 50 ng/mL TPO, and 1 μg/mL LDL. Small molecule inhibitor UM729 (500 nM) was also added to the media where indicated. For control conditions, 1 or 3 units of media were added every 24 hours to mimic the feeding scheme of the fed-batch system. For conditions using the PID control algorithm, media was added as indicated by calculations. For “no dilution” conditions, a complete media exchange was performed every four days.

### Phenotypic analysis

For model development, surface marker staining was performed with conjugated human antibodies: CD34, CD38, CD45RA, CD90, CD49f, CD10, CD135, CD7, CD3, CD19, CD41, CD235a, CD11b, CD16, CD33, CD14 (BD Biosciences). Surface marker staining for validation experiments was performed with conjugated human antibodies: CD34, CD45RA, CD90 (BD Biosciences). All samples were analyzed on a FACSCanto or FACS LSRFortessa flow cytometer (BD Biosciences).

### Statistical Analysis

Statistical significance was calculated using a two-tailed Student’s t test, assuming unequal variance, a paired Student’s t-test, or a one-way ANOVA. Error bars represent the standard error of 3 biological samples or the standard deviation of 100 or more in silico replicates. Asterisks indicate statistical significance between indicated samples.

## Results and Discussion

**Fig 1:**
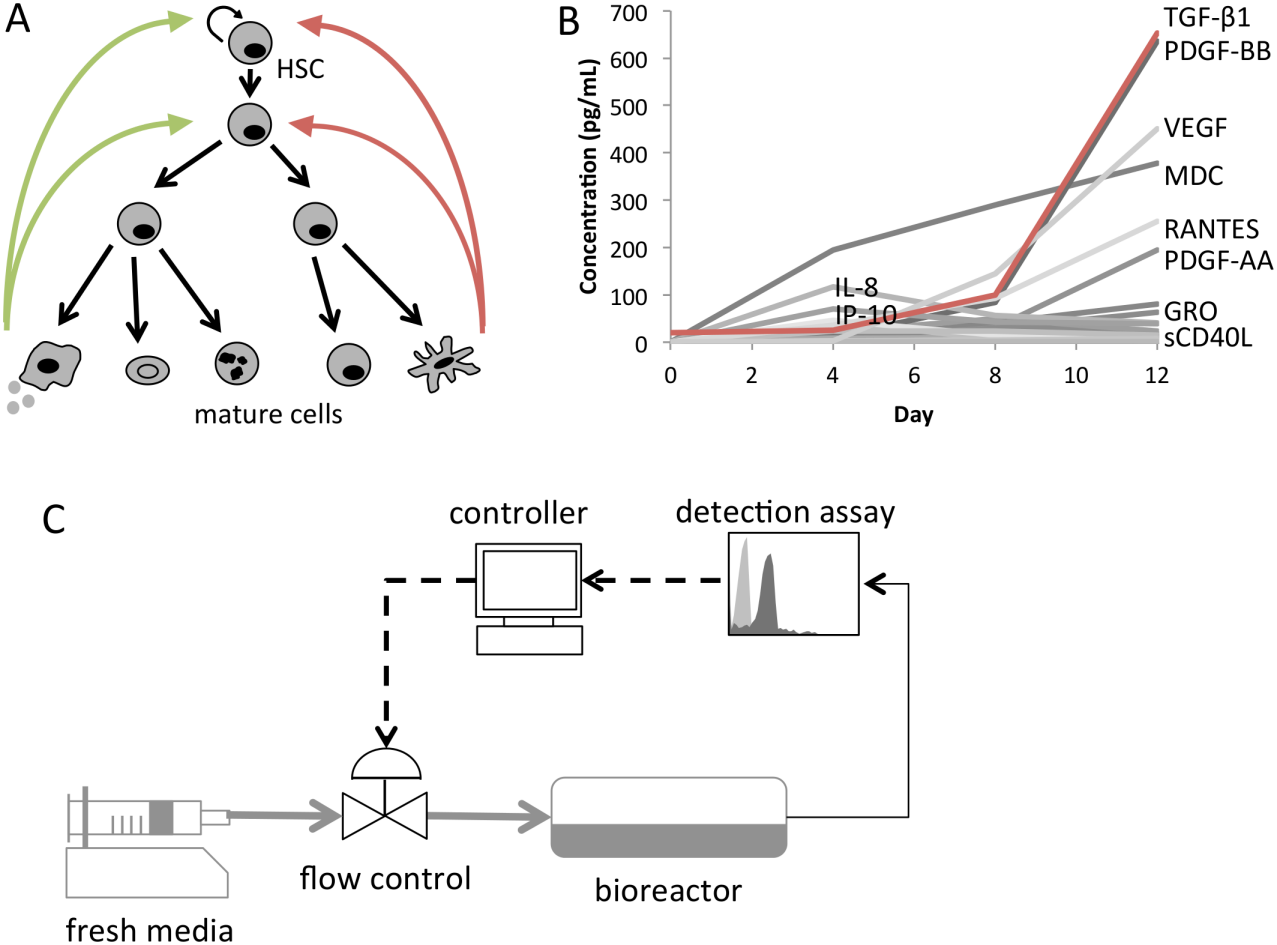
Soluble factors regulate HSPC expansion. **[A]** Mature cell phenotypes secrete factors that positively (green) and negatively (red) regulate stem and progenitor expansion **[B]** Cytokines and chemokines accumulate in fed-batch culture negatively regulating HSPC expansion. Data represents mean concentration of n = 2 (Luminex) or n = 4 (ELISA) biological replicates in fed batch culture using a linear dilution scheme (D = 1). **[C]** Schematic of the fed-batch bioreactor system with a feedback controlled flow controller. The TGF-β1 concentration is determined by measuring the concentration of the representative factor, LAP, using the microbead detection assay. The TGF-β1 concentration is input to the controller which adjusts the media flow rate to control the concentration of TGF-β1, allowing for real-time control of soluble factor accumulation.

During culture, cell fate decisions are governed by a complex network of secreted factors, each of which can have a positive or negative effect on the stem and progenitor populations, as depicted in Fig 1A. The objective of this study was to design a feedback control system to regulate the concentrations of these factors, thereby improving HSPC expansion. To facilitate controller design, a single target factor was used to represent the effect of the milieu. As shown in Fig 1B, TGF-β1 accumulates at high concentrations in a pattern that is characteristic of many other inhibitory factors. It has previously been shown to have a strong inhibitory effect on the expansion of HSPCs and is secreted by all hematopoietic phenotypes in culture [8, 23, 24]. It was therefore chosen as the representative factor for the feedback control system.

A microbead immunoassay was used to quantify the level of secreted TGF-β1 for input to the controller. In culture, TGF-β1 accumulates in culture in latent form, requiring an activation step to measure with antibody detection [25]. To avoid this step, thereby reducing assay time, the latency-associated peptide (LAP) can be used as a surrogate for TGF-β1. LAP binds strongly to TGF-β1 to form the small latent complex and its concentration is strongly correlated to that of TGF-β1 [20, 26].

An empirical model was developed to describe the relationships between TGF-β1 and cell expansion. The model was then used to design a feedback controller, using LAP concentration as input and giving dilution as output. The controller was then combined with the microbead assay and fed batch bioreactor system to form a closed loop bioprocess, as depicted in Fig 1C.

### Model Development

An empirical model of hematopoietic cell culture was developed to describe the relationships between the secreted factor of interest, TGF-β1, and the expansion of hematopoietic cell populations in 3-factor culture conditions. The model training data was obtained by performing cord blood expansion experiments with 3 dilution schemes. A ‘no dilution’ (D = 0) scheme, wherein a complete media exchange was performed every 4 days, and two fed-batch linear dilution schemes (D = 1 and D = 3), wherein cultures were seeded in one unit of media and one or three units of media were added every 24 hours [8, 13]. TGF-β1 concentrations were measured using ELISA every four days. At the same time, fourteen blood cell phenotypes, known to be present in culture, were defined and quantified using flow cytometry; the definitions for these cell populations can be found in S1 Table. The cell types were placed into 6 phenotype groups according to growth patterns in fed-batch culture. Average gene expression values for TGF-β1 were obtained for each phenotype group from microarray data, accessible at NCBI’s Gene Expression Omnibus (GEO) through accession numbers GSE42414 and GSE24759 (S2 Table). Data from these two sets were analyzed independently as previously described [6]. Averaged gene expression signals were calculated for TGF-β1 probes according to Entrez gene identifiers. The average expression signals were normalized between the sets using the ‘proB’ (CD34^+^CD10^+^CD19^+^) phenotype included in both data sets. These expression signals were then averaged for each of the 6 groups.

**Fig 2:**
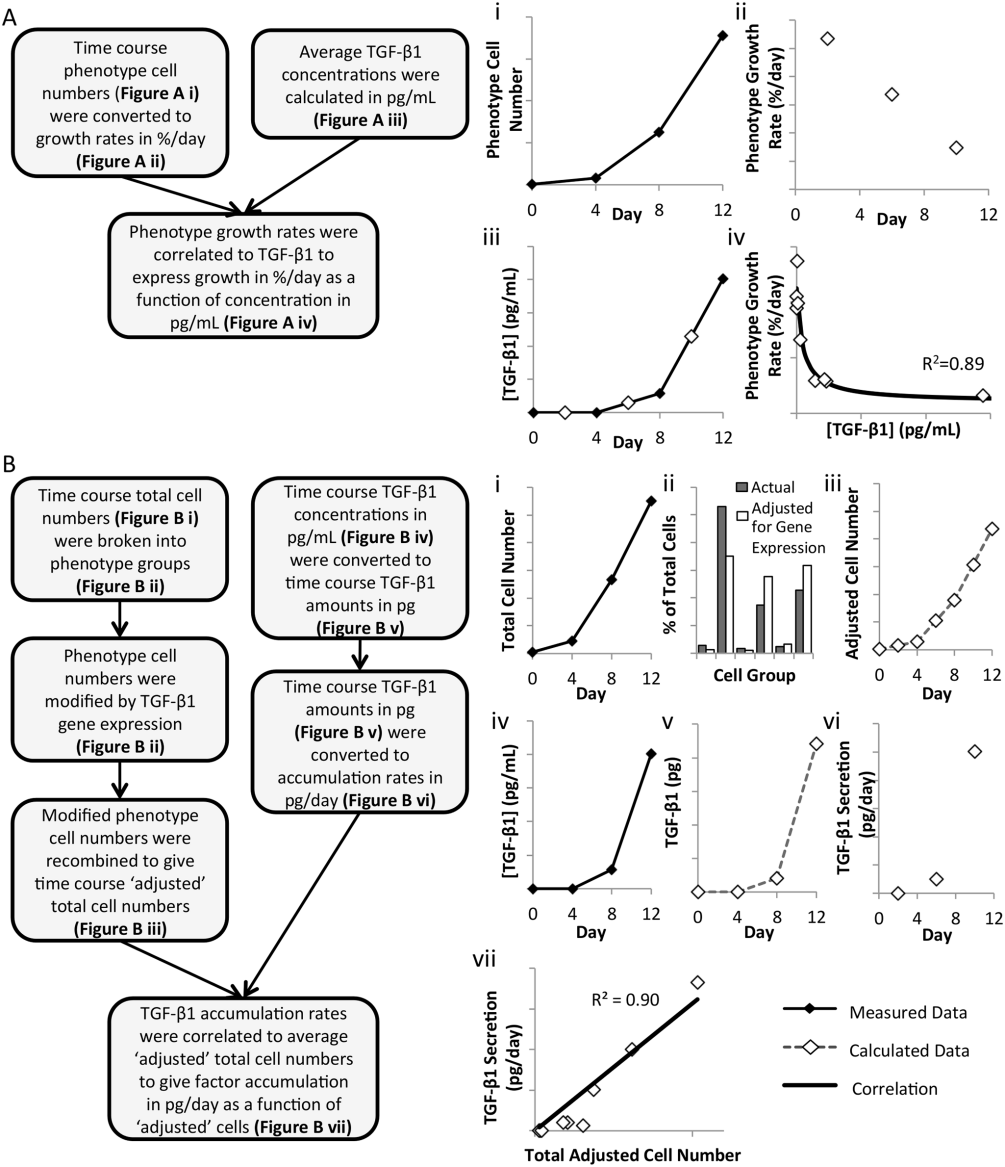
Empirical correlations were used to develop the model. **[A] (i)** Cell numbers for each phenotype group are converted to **(ii)** growth rates, as % change per day. **(iii)** Average TGF-β1 concentrations are calculated from the time course data. **(iv)** These midpoints (day 2, 6, 10) are combined for all three media dilution rates and correlated to give growth rates (%/day) as a function of TGF-β1 concentration (pg/mL). **[B] (i)** Total cell numbers are categorized into phenotype groups and **(ii)** adjusted by TGF-β1 gene expression values to reflect each phenotype’s relative contribution to TGF-β1 accumulation. **(iii)** The new adjusted cell numbers are summed to give a total ‘adjusted’ cell number. Separately, **(iv)** TGF-β1 concentrations (pg/mL) are converted to **(v)** TGF-β1 amounts (pg) using the culture volume (mL). **(vi)** This is converted to secretion rates, in pg/day. **(vii)** These midpoints (day 2, 6, 10) are combined for all three dilution rates and correlated to give TGF-β1 secretion (pg/day) as a function of total ‘adjusted’ cell number.

The time-course phenotype and TGF-β1 data were analyzed and correlated to express cell expansion as a function of factor concentration and factor accumulation as a function of cell number. Fig 2A outlines the stepwise data analysis performed for each phenotype group to correlate cell growth rate as a function of TGF- β1 concentration in the culture media. The time-course data for each phenotype group was used to calculate cumulative growth rates, as the percent change in cell number per day as a function of the current number of cells in that group. This was repeated for each of the three dilution schemes. These values were correlated to the corresponding time-course TGF-β1 concentrations using the CurveFit toolbox in Matlab 2012a (Mathworks, Natick, MA). Exponential functions were used when the phenotype growth rate exhibited a negative dose response to TGF-β1 concentrations. The remaining phenotypes were fitted with splines. These growth rate-concentration correlations are shown in S1 Fig for 5 phenotype groups and the 4 phenotypes in the sixth group. The data analysis steps performed to establish a relationship between TGF- β1 secretion rate and total cell number are outlined in Fig 2B. Again, for each of the three dilution schemes, the rates of TGF-β1 accumulation in pg/day were calculated from the time-course concentrations. The measured cell numbers in each phenotype group were modified by their group’s average gene expression value to obtain an ‘adjusted’ cell number, reflective of the group’s contribution to TGF-β1 accumulation. This was correlated to the factor accumulation to calculate a secretion rate in pg/‘adjusted’ cell per day.

**Fig 3:**
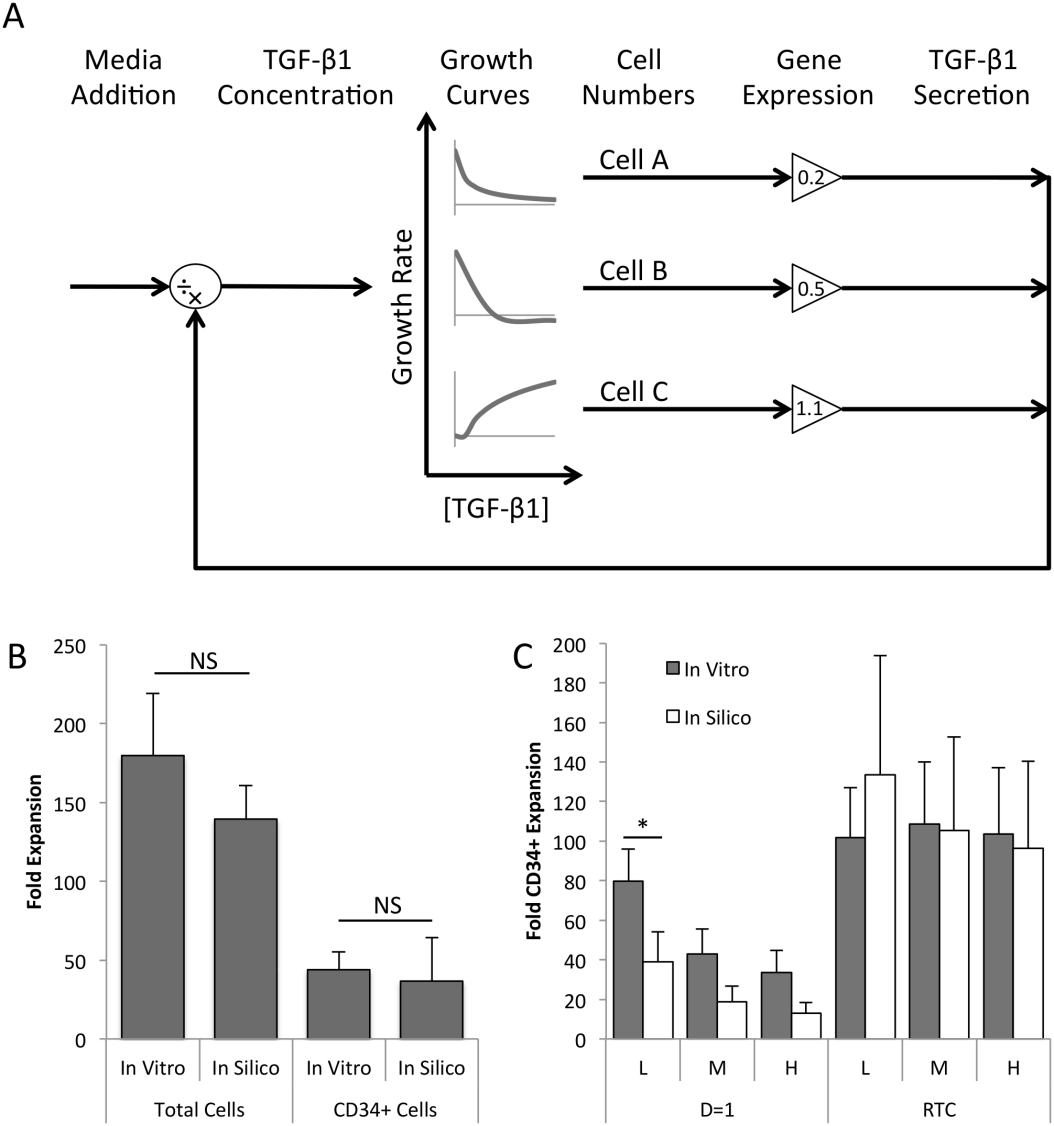
Model structure and performance. **[A]** Cumulative growth curves are defined for each phenotype group to calculate expansion as a function of TGF-β1 concentration. Cell numbers are adjusted by TGF-β1 gene expression values, which increase or decrease each phenotype’s relative contribution to factor accumulation as illustrated, to predict TGF-β1 secretion rates and accumulation in the media. The TGF-β1 concentration calculated using the culture volume, where the concentration is controlled by media addition, specified as a flow rate. This flow rate can be specified as a function of time or can be calculated through an external feedback control loop. **[B]** The model (n = 10) recapitulates average in vitro (n = 3) total cell and CD34^+^ cell expansion at day 12 of culture, as well as population level variability. Error bars represent standard deviation. **[C]** The model (n = 100) accurately predicts HSPC expansion observed during the RTC experiment (n = 3). Cells were seeded at low (L), medium (M), and high (H) densities, and cultures were diluted using a linear scheme (D = 1) or the simple feedback algorithm (RTC). Adapted from Csaszar et al. 2014 [20]. * *p*<0.05.

These resulting correlations were incorporated into a Simulink model to perform a continuous dynamic simulation of fed-batch culture (Fig 3A). The model describes the inherent feedback loop of cell expansion and factor accumulation, and allows for perturbations in the form of dilution (altering the factor concentration). The current culture volume serves as input to the model. This is used to calculate the TGF-β1 concentration (TGF-β1 ÷ volume). The model uses this TGF-β1 concentration to calculate the growth rate for each phenotype group from the growth rate-concentration correlations and updates their respective cell numbers (cells × growth rate, integrated over time). These cell numbers are modified by the gene expression values (cells × gene expression) and summed to find the ‘adjusted’ cell number. From this value, the net TGF-β1 secretion rate is calculated using the secretion rate-cell number correlation (‘adjusted cell’ × secretion rate) and integrated to give the total amount of factor. The feedback loop is closed to complete the model of the feedback regulation inherent to hematopoietic cell culture. This feedback regulation is perturbed only by changes in the culture volume, the only method of culture manipulation that is applied.

The Simulink model is accompanied by a Matlab script to specify the input conditions, including initial cell numbers in each group. This starting population is highly variable, even after cell selection, and has been correlated with the achievable expansion in culture [20]. To incorporate this variability, the model incorporates known distributions of surface markers to define the input population. Specifically, distribution curves for two surface markers, CD34 and CD38, were obtained for CD34^+^ or Lin^-^ selected cord blood cells. When incorporated into the model, these two surface markers simulate unique biological replicates, and recapitulate the variability observed on a population level. The distribution curves used in the model are shown in S2 Fig.

The model was first validated using the training data set, and was found to accurately replicate both total cell and CD34^+^ cell expansion after 12 days of culture (Fig 3B). The model was then tested by comparing model outputs to independently generated, previously reported findings using a linear dilution scheme and a real-time control (RTC) system [20]. Fig 3C shows that the model accurately recapitulates the trends observed in this study, and captures the effects of the feedback control system. This confirmed the model could be used to simulated feedback control conditions.

### PID Controller Design

**Fig 4:**
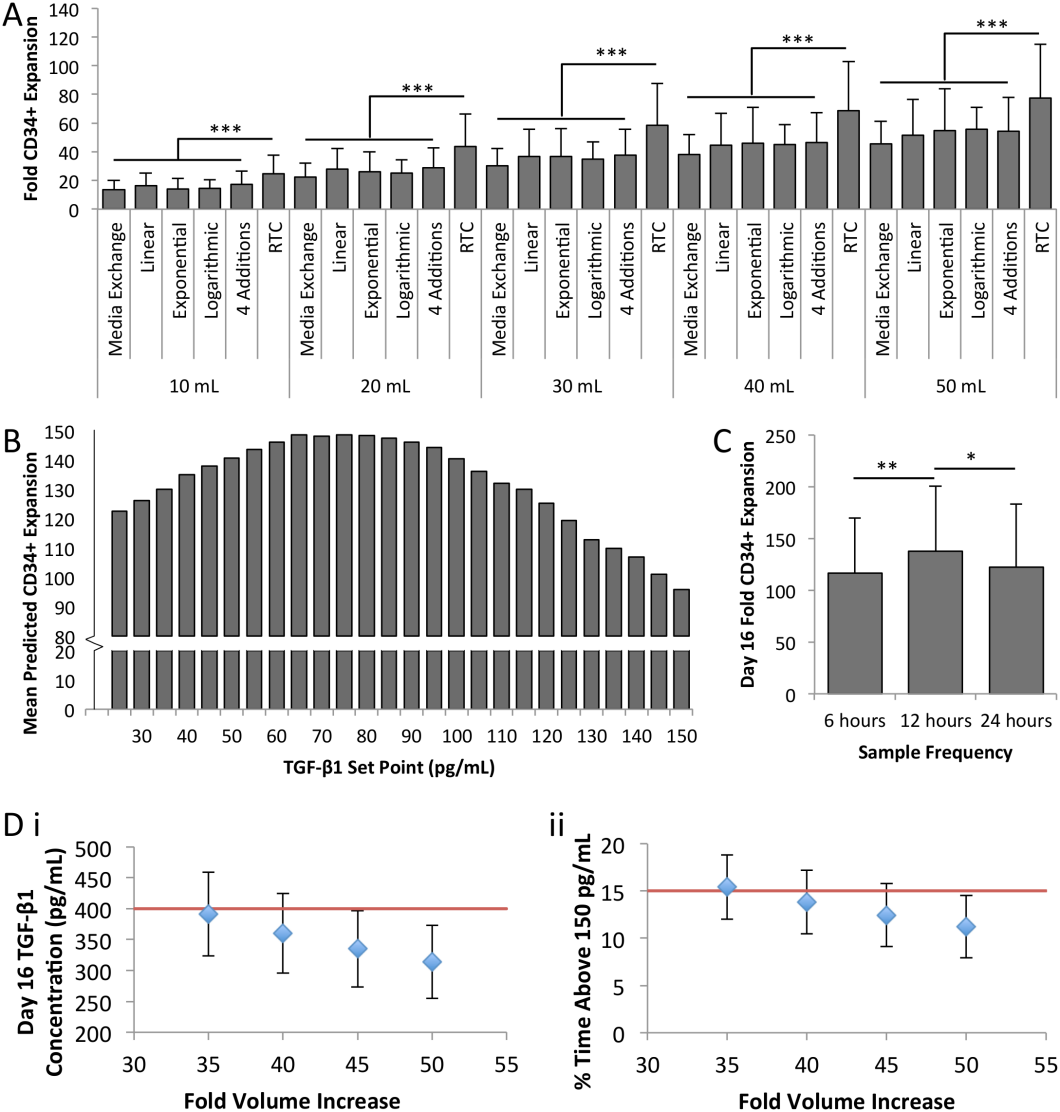
Model predicts a PID controller is the optimal media delivery method. **[A]** Different feeding strategies were investigated in silico. When normalized to day 16 total media requirements, a simple algorithm (RTC) significantly outperforms other media delivery strategies for CD34^+^ expansion (n = 100). **[B]** The PID controller performs optimally at a set point of 85 pg/mL for any fold volume increase (n = 100). **[C]** CD34^+^ expansion is improved using 12 hour sampling over 6 hour or 24 hour sampling (n = 100). **[D]** A 50-fold volume increase using the PID controller is the minimum fold volume increase to meet both concentration constraints (<400 pg/mL TGF-β1 on day 16 and <15% of time above 150 pg/mL) (n = 100). * *p*<0.05, ** *p*<0.01, *** *p*<0.001.

Next, we used the model to confirm that feedback control could be an efficient media addition strategy. By defining the rates of volume change in the model, different dilution strategies could be simulated. The RTC feedback algorithm was compared to media exchange (“no dilution”), linear, exponential and logarithmic dilution schemes [20]. Fig 4A shows that when these results are normalized to total media consumption, CD34^+^ expansion is improved with the RTC algorithm, indicating that feedback control is a significantly more efficient way to deliver media in a fed-batch culture system.

We hypothesized that optimizing the controller design and TGF-β1 concentration set point would further improve the efficiency of feedback dilution for fed-batch culture. The controller uses TGF-β1 concentration as its input, and gives a dilution rate as the output. We used the empirical model to design a PID control algorithm to optimize CD34^+^ expansion and media requirements over 16 days of culture. This time point was selected as it reflects the time point where the RTC scheme has measurable benefits [20]. An efficient dilution strategy should maximize output HSPCs per mL of media used. To ensure this strategy can be scaled to produce clinically relevant cell numbers, these parameters were normalized. We defined the dimensionless parameter, volumetric efficiency, to be the ratio of fold CD34^+^ expansion (day 16 CD34^+^ cell number/day 0 CD34^+^ cell number) to fold volume increase (day 16 volume/day 0 volume). This volumetric efficiency parameter reflects the cost efficiency of the bioprocess, reflecting the productivity (HSPC expansion) per dollar of input resources (media), and was used to compare the efficiency of different media dilution control profiles. The input cell density was held constant at 100,000 cells/mL. The initial culture volume used in the model is 1 mL. Our aim was to design a controller that maximizes volumetric efficiency.

Given that our model of hematopoietic cell culture is non-linear and the process is not at steady state, the PID control algorithm was manually tuned using the model. A discrete PID controller has 4 parameters: a proportional term (P), integral term (I), derivative term (D) and filter term (N). A set point and sampling frequency (T_s_) are also used. For an unsteady state system, the integral term is set to zero because incorporating cumulative error does not improve controller performance. The parameters were tuned iteratively, beginning with a proportional-only controller. This exercise was performed for a range of fold volume increases (16–65), equivalent to linear dilution rates of 1 to 4. The fold volume increases were set using a saturation limit that turned the controller off when the volume limit had been reached. The factor accumulation pattern is consistent across conditions up to the time at which the controller is turned off. Therefore, it was found that the controller parameters converged to the same values in each repetition of the exercise, resulting in a single, optimal controller. Fig 4B, 4C and S7 Fig show the effect of altering the set point and sampling frequency of the controller on population level expansion.

When we investigated the effects of this controller *in silico*, we observed a limitation in our model wherein maximizing the volumetric efficiency with no other constraints predicted that volumetric efficiency decreases with increasing fold volume increases. However, extended periods of no dilution or media exchange would result in the accumulation of inhibitory secreted factors resulting in rapid cell differentiation and poor cell growth. For example, when the lowest fold volume increase was used, the model predicted the controller would be turned off at day 6.5±0.5 days (n = 100) and the factor concentration was predicted to reach very high levels, 952±114 pg/mL. The model calculates the growth rate of each phenotype independently. At high concentrations of TGF-β1, the model predicts rapid expansion in the mature cell phenotypes, but this is not necessarily accompanied by a loss of progenitor cells, as would be expected during differentiation. Because of this, the model is likely to overestimate CD34+ expansion under these conditions. To avoid these rapid differentiation conditions, additional constraints were incorporated into the model to determine the optimal fold volume increase. Previous fed-batch experiments were analyzed to identify conditions that resulted in rapid differentiation [13, 20]. S3 Fig shows that the final TGF-β1 concentration and the cumulative time the cells were exposed to a high concentration of TGF-β1 were most predictive of the loss of the CD34^+^ phenotype. New optimized conditions were implemented with the controller to ensure the TGF-β1 concentration would not exceed 150 pg/mL for more than 15% of the total culture time, nor would it exceed 400 pg/mL by the endpoint. When combined with the optimal controller, a minimum fold volume increase of 50 (equivalent to a linear dilution rate of approximately 3) meets these conditions with at least 85% of replicates (1 standard deviation), as shown in Fig 4D. The resulting PID controller has a TGF-β1 set point of 85 pg/mL, a sampling frequency of 12 hours, and a maximum fold volume increase of 50.

### Model Predicts Feedback Control Using Controller Enhances CD34^+^ Expansion

**Fig 5:**
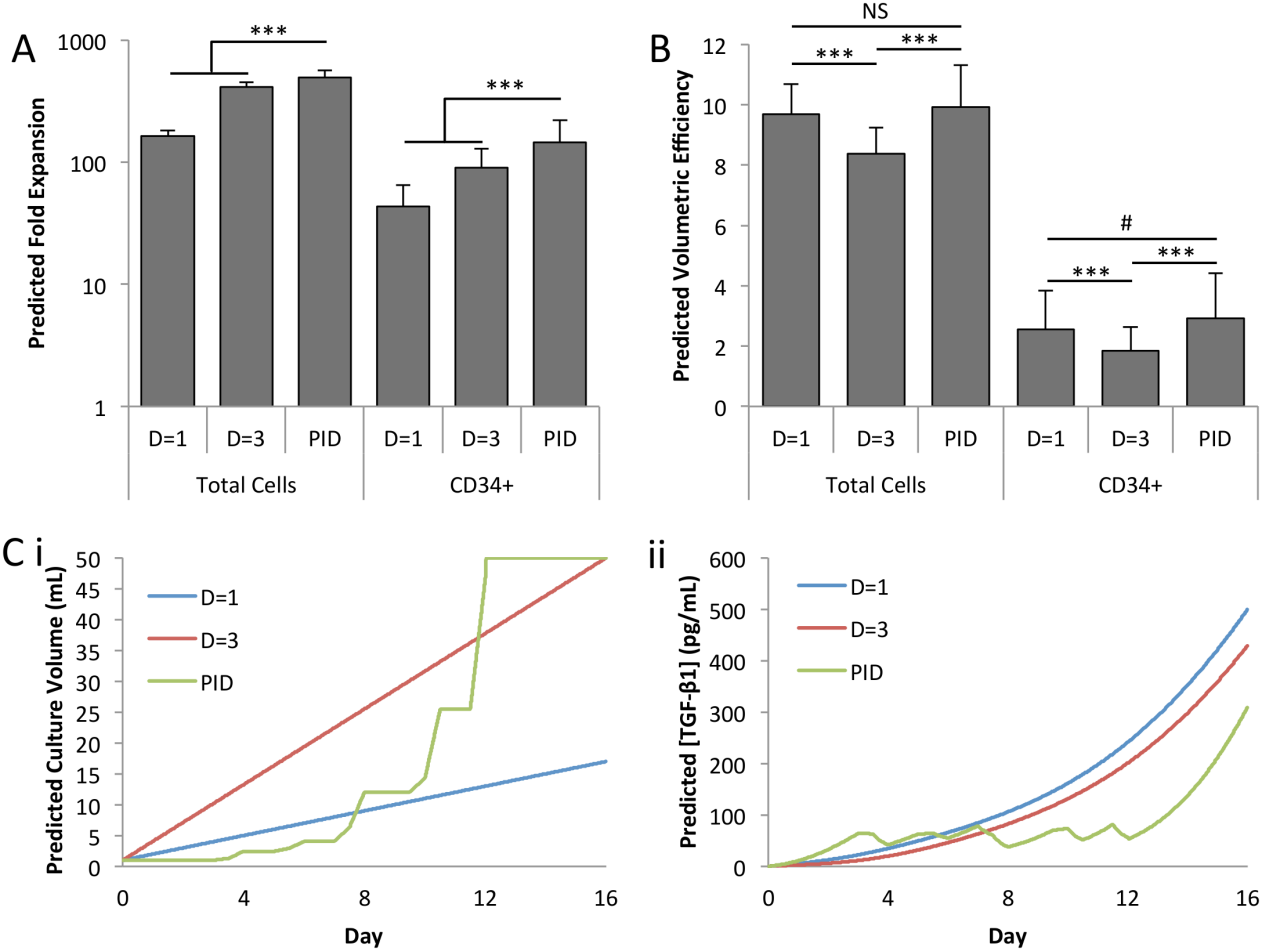
Model predicts optimal PID controller improves expansion by maintaining low factor concentration. **[A]** Model predicts significant improvement in both total cell and CD34^+^ cell expansion over both linear dilution at one unit per day (D = 1) and volume matched linear dilution (D = 3). **[B]** Volumetric efficiency (fold expansion/fold volume increase) is recovered to D = 1 levels by using the PID controller. **[C]** Predicted **(i)** volume and **(ii)** concentration trajectories for a representative sample. # *p*<0.1, *** *p*<0.001, NS *p*>0.1.

The optimized control strategy was implemented in silico to predict the achievable cell expansion in fed batch culture. Fig 5A shows that the PID controlled cultures were predicted to improve both total cell and CD34^+^ cell expansion over two linear dilution controls. The D = 1 dilution scheme is the result of previous optimization of fed-batch culture [13], with one unit of media added each 24 hours, and results in a 16-fold volume increase over 16 days of culture. The D = 3 dilution scheme adds 3 units of media each 24 hours, and provides a volume-matched control for PID control. A 146-fold average HSPC expansion was predicted using the PID controller compared to 90-fold for the D = 3 dilution scheme. Fig 5B demonstrates that the predicted volumetric efficiency of the D = 3 linear dilution scheme was significantly lower than that of the previously optimized D = 1 control condition. The use of the PID controller overcame this, resulting in the same (with respect to total cells) or higher (with respect to CD34^+^ cells) volumetric efficiency as the D = 1 condition. The increased media requirement for PID control is justified by the economic benefits of increased expansion with equivalent efficiency when compared to the standard D = 1 conditions. Sample model outputs for culture volume and TGF-β1 concentration can be seen in Fig 5C, with the full ranges of possible outputs shown in S5 Fig. As previously described, the rate of media addition was determined by the controller until the volume saturation limit is reached, after which the TGF-β1 concentration continues to increase.

### Feedback Control Facilitates Rapid Expansion of CD34^+^ Cells

We next implemented the PID controller in the fed batch culture system to validate our model predictions. As in the model, our previously described optimal linear dilution strategy (D = 1, 16-fold volume increase) and a volume matched linear dilution strategy (D = 3, 50-fold volume increase) were used as controls. In the model, the derivative term of the PID controller was calculated using a Euler forward approximation of the difference. To facilitate the manual implementation used in this study, the controller output (media flow rate) was modified to give bolus media delivery. The derivative was also calculated using a less computationally intensive backwards approximation of the difference. S4 Fig highlights the predicted effects of these changes. Notably, the use of the PID controller in this configuration is predicted to still outperform both the linear dilution schemes.

**Fig 6:**
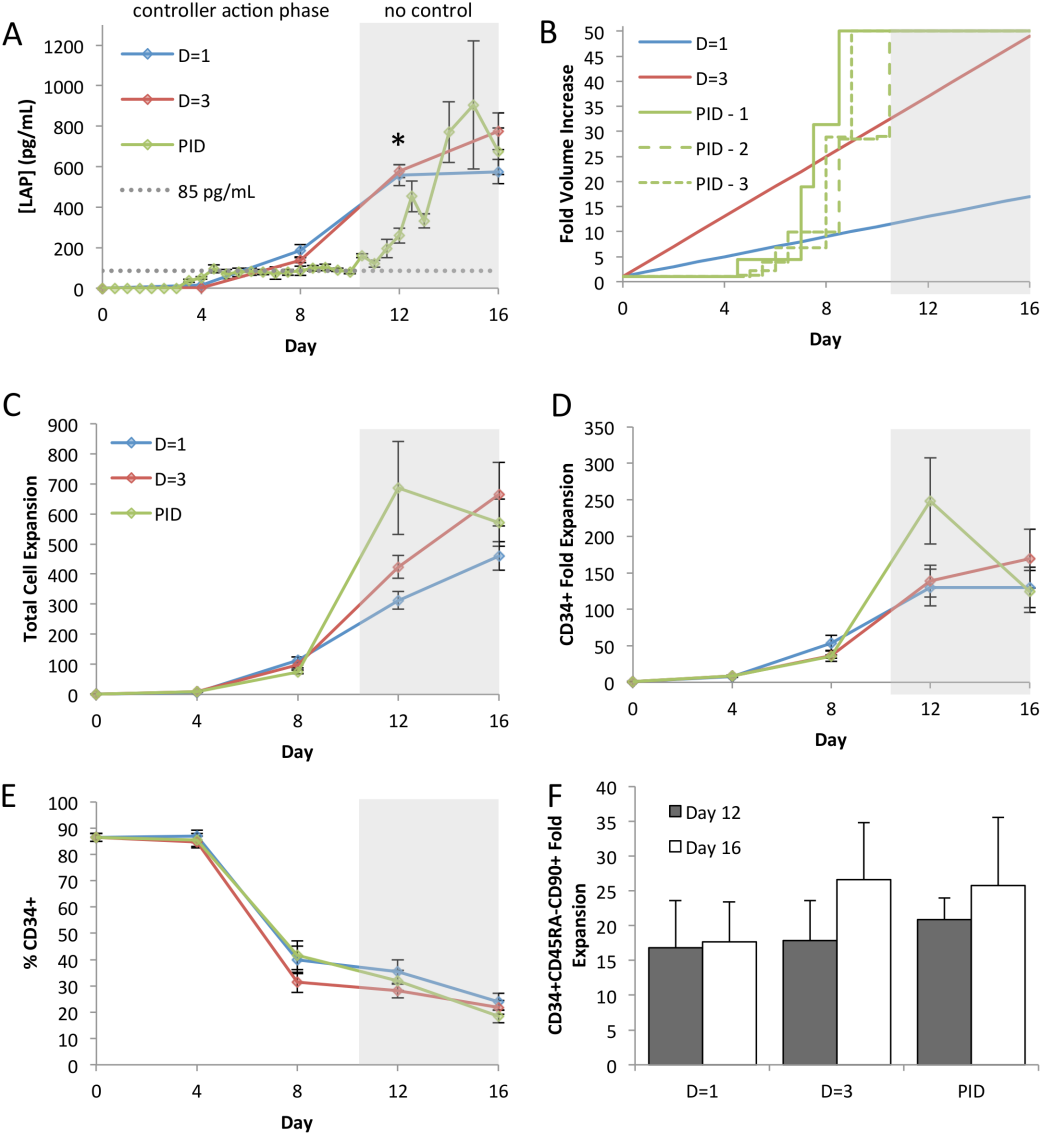
PID controller facilitates rapid cell expansion. **[A]** Average LAP concentration time course demonstrates that the PID controller maintains a lower factor concentration (n = 3) than linear medium dilution strategies during the controller action phase. **[B]** Volume trajectories for D = 1, D = 3 and 3 PID controlled samples shows controller action between days 5 and 10. **[C]** Total cell expansion compared between dilution strategies. PID control outperforms both linear dilution schemes at day 12 (n = 3). **[D]** CD34^+^ cell expansion compared between dilution strategies. PID controller outperforms both linear dilution schemes at day 12, with the effect lost by day 16 (n = 3). **[E]** Surface marker analysis of CD34^+^ frequency during culture shows rapid differentiation between day 12 and 16 with PID control (n = 3). **[F]** Despite net loss of both total and CD34^+^ cells, day 16 expansion of HSC-enriched population CD34^+^CD45RA^-^CD90^+^ is not adversely affected (n = 3). * *p*<0.05.

In fed-batch culture, the PID controller maintained the LAP concentration at 85 ± 20 pg/mL during the controller action phase (days 4.5 through 10.5) before reaching the media volume limit as depicted in Fig 6A and 6B. Fig 6C and 6D shows that at day 12 this resulted in enhanced total cell and CD34^+^ cells expansion compared to both control conditions. Although the PID conditions used more media at this time point compared to the volume matched control (50-fold volume increase and 34-fold volume increase, respectively), the volumetric efficiency was moderately improved at this time point (5.0±1.2 compared to 4.1±0.6). The D = 1 dilution scheme was more efficient at this time point (10.8±2.1). However, beyond day 12 we saw a rapid loss of CD34^+^ cells, as seen by a significant decrease in %CD34^+^ cells between day 12 and 16 (Fig 6E). The volumetric efficiency of the PID conditions also decreased, rendering it less efficient than the linear dilution schemes (2.5±0.6, 8.1±1.7, and 3.7±0.9 for PID, D = 1, and D = 3, respectively). These effects coincide with the accumulation of LAP in the last 6 days of culture during which no dilution was performed, providing an internal control for our PID system. This controller action phase was shorter than the model predicted, and can be attributed to the non-linearity of hematopoietic cell culture, as the model does not incorporate temporal changes in phenotype behavior. To verify that the PID controller was also facilitating the expansion of more primitive cells the HSC enriched population CD34^+^CD45RA^-^CD90^+^ was assessed [27]. Fig 6F shows that we found the PID conditions expanded these cells as well as the linear controls at both day 12 and day 16, suggesting that the deleterious effects of the late accumulation of LAP are delayed in this more primitive population.

### Feedback Control Achieves Expansion Comparable to UM729

**Fig 7:**
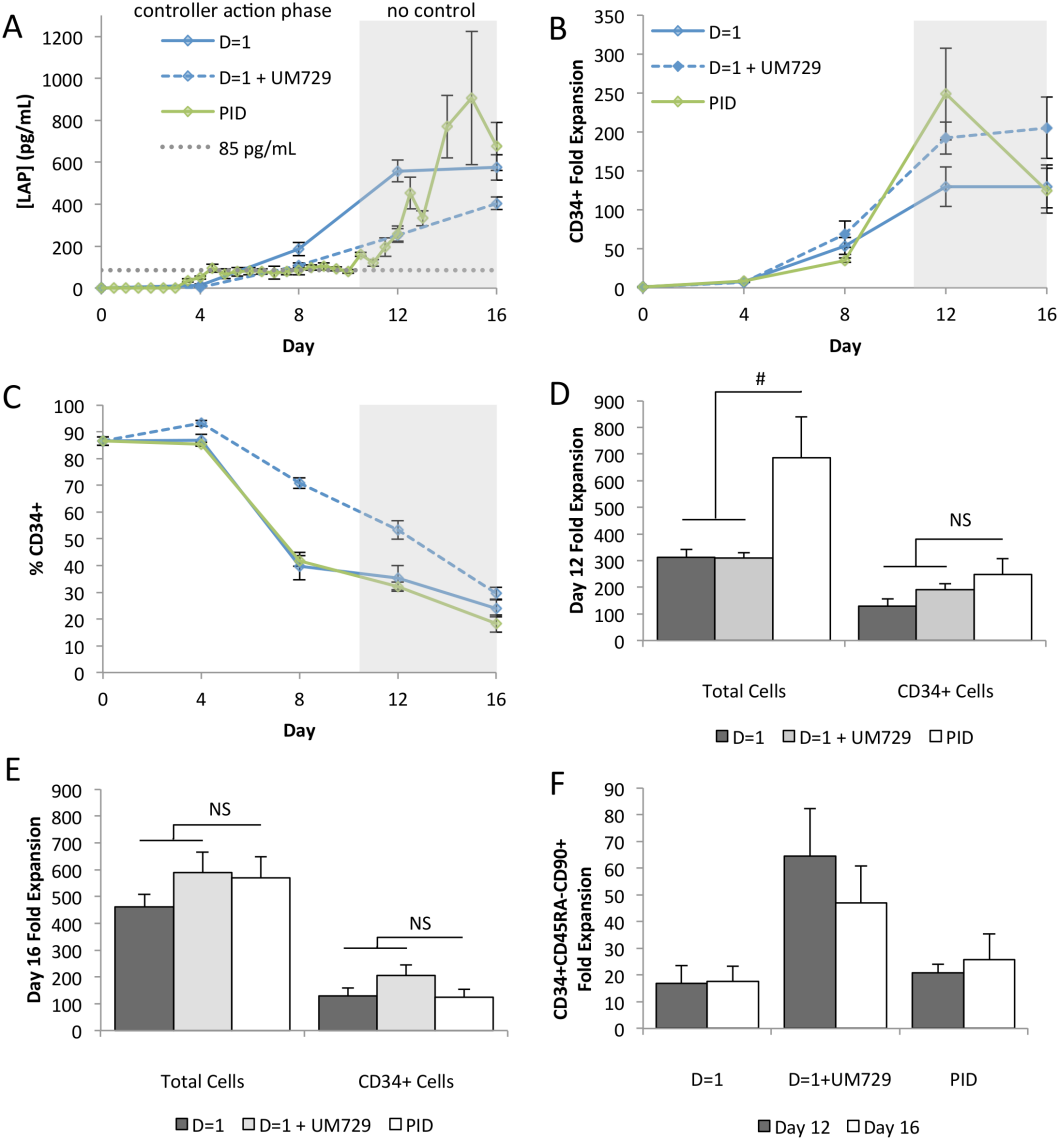
PID control is an effective alternative to supplementation with UM729. **[A]** Our PID feedback controller has a similar effect on the concentration of LAP as UM729 addition with the PID controller conditions have a significantly higher concentration only at day 4 (n = 3). **[B]** Time course CD34^+^ expansion shows that PID control performs at least as well as D = 1+UM729 at day 12. **[C]** UM729 inhibits differentiation, resulting in a higher proportion of CD34^+^ cells beyond day 4. Both conditions have a significant loss of this phenotype between days 12 and 16 (D = 1+UM729 p = 0.007, PID p = 0.047) (n = 3). **[D]** At day 12, PID control moderately outperforms D = 1^+^UM729 with regards to total cell expansion. CD34^+^ cell expansion is equivalent (p = 0.444) (n = 3). **[E]** When culture time is extended to day 16, PID control and D = 1+UM729 remain equivalent (total cells p = 0.871, CD34^+^ cells p = 0.178) (n = 3). **[F]** Higher expansion of the HSC-enriched population, CD34^+^CD45RA^-^CD90^+^, is observed in D = 1+UM729 conditions at both day 12 and day 16, though the difference is not significant. # *p*<0.1 NS *p*>0.1.

We have recently described the impact of a small molecule, UM729, on UCB expansion [15]. As this molecule acts (in part) by limiting differentiation (and thus negative factor accumulation), we next wanted to compare cultures performed with and without our control strategy, to those with and without UM729. As shown in Fig 7A, PID control maintains low levels of LAP most similar to D = 1+UM729. The proportion of CD34^+^ cells is better maintained in cultures supplemented with UM729 (Fig 7C). Despite this, at day 12, the optimal end point for UM729 supplemented cultures, PID control alone performed at least as well as D = 1+UM729. Total cell expansion is moderately improved, and CD34^+^ cell expansion is equivalent (Fig 7D). This difference in total cell expansion can be attributed to the effect of the UM729 molecule, which has been shown to reduce the frequency of mature phenotypes in culture. However, the increased number of more mature blood cells produced using PID control may offer a clinical advantage to HSC transplant recipients [28]. Although there is rapid loss of both total and CD34^+^ cells in PID conditions when cultures are extended to 16 days, PID control continues to produce outputs equivalent to D = 1+UM729 (Fig 7B and 7E). When we looked at the CD34^+^CD54RA^-^CD90^+^ HSC enriched population in Fig 7F, we found that PID control output was lower than D = 1+UM729, consistent with the impact of this molecule on the most primitive progenitor sub-set.

We investigated whether combining the PID controller with UM729 would have a synergistic effect. Though PID control and UM729 extended the controller action phase and maintained low levels of LAP through to the end of culture, it had only modest effects on cell expansion (S6 Fig). The inefficacy of the PID controller combined with UM729 can be attributed to the effects of the molecule. UM729 inhibits differentiation, and with fewer mature phenotypes in the culture, the concentrations of negative factors are reduced accordingly. While PID control further reduced the concentration of LAP compared to linear dilution, the magnitude of this difference is much smaller than observed in 3-factor cultures and is insufficient to produce a measurable effect on cell outputs. We hypothesize, based on *in silico* modeling, that a lower set point will enable synergistic improvements with the combination of the two technologies. The lower detection limit of the microbead immunoassay is not sufficient to accurately implement a lower set point, requiring further ongoing technology development.

## Conclusions

The development of a feedback control system for secreted factors is an important step in developing a patient- or sample-specific bioprocess to produce personalized cell therapy products. When used for the ex vivo expansion of HSPCs, further development of this bioprocess may enable the use of UCB for transplantation in adult patients. In this study, we have developed a new empirical model of hematopoietic cell culture and used it to design a feedback control strategy for regulating concentrations of inhibitory secreted factors. This feedback control strategy was used to predict the maximum volumetric efficiency that could be achieved in our HSPC culture, resulting in a more cost efficient bioprocess. We experimentally validated the model predictions and found that CD34^+^ cell expansion was improved at day 12, but that the culture conditions were no longer supportive for progenitor cell populations by day 16. We confirmed that this feedback controller supported progenitor but not stem cell expansion at similar levels as the current best in class bioprocess that combines fed-batch dilution and the small molecule UM729.

Our modeling approach, which applied a mechanistic foundation to an empirical model, enables the previously uninvestigated use of feedback control for stem cell production. By incorporating the known relationships between cell expansion and secreted factors, the model is able to describe the effects of feedback-modulated dilution in fed-batch culture. The utility of the model was demonstrated by designing a PID controller that efficiently expanded HSPCs in 3-factor culture conditions. This scalable feedback control offers a cost effective bioprocess by producing equivalent or better HSPC outputs in less time for the same input media volumes. Additional online optimization of culture duration and media usage could further improve the process.

Furthermore, the use of PID control for a single factor did not have a measurable effect on the variability observed between samples. The signaling network that regulates expansion can be represented by the inhibitory factor TGF-β1, as demonstrated by the ability of the single factor model to make accurate predictions. However, the complexity of the intercellular signaling network would suggest that combining the PID controller with additional feedback loops would be beneficial. Modifying the controller to use multiple endogenously produced factor concentrations as input, or incorporating additional controllers would allow for the control of other inhibitory factors through dilution, particularly those with very different accumulation patterns. Controlling the concentration of the positive factors supplemented in the culture would ensure these molecules have a beneficial event throughout culture. Together these would create more tightly regulated conditions, thereby reducing variability and improving the robustness of the bioprocess.

The development of an improved biosensor, such as one using an electrochemical signal, rather than fluorescent, would allow further investigation of both PID control in combination with UM729 and feedback control of additional secreted factors. Functionalized nanostructured microelectrodes are a promising alternative, as they have been shown to be able to detect proteins at low concentrations, can be placed “on chip” for integration into a closed bioprocess, and can be multiplexed to detect multiple secreted factors at one time [29].

Further validation of the process will also be required to confirm the function of the output cells. HSCs are needed for long-term engraftment, and are best quantified using in vivo transplantation assays. The surface markers used to assess the phenotypes present in culture are a surrogate for function, and the correlations between surface markers and function may be lost during the culture process [30]. The outputs of the next-generation bioprocess should be evaluated using serial transplantation at limiting dilutions in NOD/Scid IL2Rγ^null^ (NSG) mice to accurately compare to the fed-batch UM171 strategy [15].

The microenvironment is responsible for HSPC cell fate decisions, and the ability to carefully control the factors governing these decisions allows for enhanced expansion and directed differentiation. We have previously shown that real-time feedback control through dilution reduces factor concentrations and improves expansion [20]. Here we have shown that this feedback control can be optimized to reduce media consumption and create a more cost effective bioprocess. Automation and further optimization of the system is expected to produce a clinically relevant bioprocess to robustly expand large numbers of HSPCs ex vivo. Extending the bioprocess to generate mature cells from the same unit of UCB, such as those required to combat post-transplantation immunodeficiency, would improve the efficacy of this therapy. The availability of personalized bioreactors, with the ability to tune the output cell composition, incorporate gene therapies, and produce competent immune cells, would make these therapies available to all who need them.

## Supporting Information

S1 Fig Growth Curves. The correlations are used to calculate the growth rates at each time step of the model. Each relates the growth rate in % change per day to the current concentration of TGF-β1. **[A]** Group 1 **[B]** Group 2 **[C]** Group 3 **[D]** Group 4, **[E]** Group 5 **[F-I]** Group 6, **[F]** NK **[G]** T **[H]** ERY **[I]** NEUT.
(TIF)

S2 Fig Model Variability. **[A]** The input cell population is highly variable, even after Lin^-^ or CD34^+^ cell selection. **[B]** This variability has an effect on cell expansion, as %CD34^+^ starting cells is positively correlated with cell expansion (adapted from Csaszar et al. 2014 [20]). **[C]** The model incorporates two distributions for **(i)** CD34^+^ and **(ii)** CD38^+^ (as % of CD34^+^) to capture this variability. The primary y-axis corresponds to the observed frequency (n = 500 in silico replicates) while ϕ is the underlying probability density function used to generate the distributions.
(TIF)

S3 Fig Controller Constraints. Conditions with a decrease in CD34^+^ cell number in the last days of culture can be differentiated from those with continued expansion **[A]** Concentration of TGF-β1 was predictive of differentiation at the end of culture, with a threshold of 400 pg/mL. **[B]** Cumulative culture times at high factor concentrations were predictive of differentiation. The threshold was set at 15% above 150 pg/mL.
(TIF)

S4 Fig Controller Calculations. **[A]** The PID controller was designed to determine a media flow rate using a forward Euler approximation of the derivative. **[B]** When the controller output is calculated as a bolus delivery of media, as during manual implementation of the controller, no change is predicted in CD34^+^ expansion (n = 100). Predicted **[C]** concentration and **[D]** culture volume trajectories for the previously mentioned representative sample highlight the differences between the media delivery methods. Manual implementation of the controller requires the use of a backwards approximation of the derivative. **[E]** The controller output is calculated at each time step *k* using equations 1–3. If *U(t*_*k*_ *)*>0, the media bolus is calculated using equation 4. **[F]** This calculation method lowers the predicted CD34^+^ cell expansion, but it remains an improvement over the linear dilution schemes (n = 100). Predicted **[G]** concentration and **[H]** volume trajectories for the representative sample highlight that backwards differentiation results in an extended controller action phase but higher average factor concentration. * *p*<0.05, *** *p*<0.001.
(TIF)

S5 Fig Predicted Ranges from Model. **[A]** The model predicts a broad range of possible volume trajectories when implemented with the PID controller. The solid black line represents the average value of 100 *in silico* replicates. The dark grey band shows the mean ± 1 standard deviation. The light grey band shows the full range of predicted values. **[B]** The model correspondingly predicts a broad range of possible TGF-β1 concentrations. **[C]** This results in a wide range of predicted CD34^+^ expansion, representative of population-level biological variability between units of cord blood.
(TIF)

S6 Fig PID control with UM729. **[A]** Average LAP concentration time course demonstrates that the PID controller maintains a lower factor concentration (n = 3) than linear medium dilution strategies during the controller action phase, though the magnitude of this difference is small compared to 3-factor conditions. **[B]** Volume trajectories for D = 1, D = 3 and 3 PID controlled samples supplemented with UM729 shows controller action is extended to day 12. **[C]** Total cell expansion compared between dilution strategies. PID control with UM729 is not significantly different from controls at day 12 or day 16 (n = 3). **[D]** CD34^+^ cell expansion compared between dilution strategies. Again, PID control with UM729 is not significantly different from controls at day 12 or day 16 (n = 3). **[E]** Surface marker analysis of CD34^+^ frequency during culture that PID control has no effect when combined with UM729 (n = 3). **[F]** PID control with UM729 does not offer any advantages for expansion of the HSC-enriched population, CD34^+^CD45RA^-^CD90^+^ (n = 3). **[G]** *In silico* modeling suggests that PID control would have synergistic effects with UM729 when implemented with a lower set point. * *p*<0.05.
(TIF)

S7 Fig Set Point Optimization. The model predicts a TGF-β1 set point of 85 pg/mL maximizes CD34^+^ cell expansion at fold volume increases of 30, 40 and 50 (n = 100).
(TIF)

S1 Table Phenotype definitions used for model development. (DOCX)

S2 Table Phenotype Groups. (DOCX)
