# Supplementary figures and images for: Proportional-Integral-Derivative (PID) Control of Secreted Factors for Blood Stem Cell Culture
Source: PLoS One. 2015 Sep 8;10(9):e0137392. doi: 10.1371/journal.pone.0137392 (PMC4562642; doi:10.1371/journal.pone.0137392)

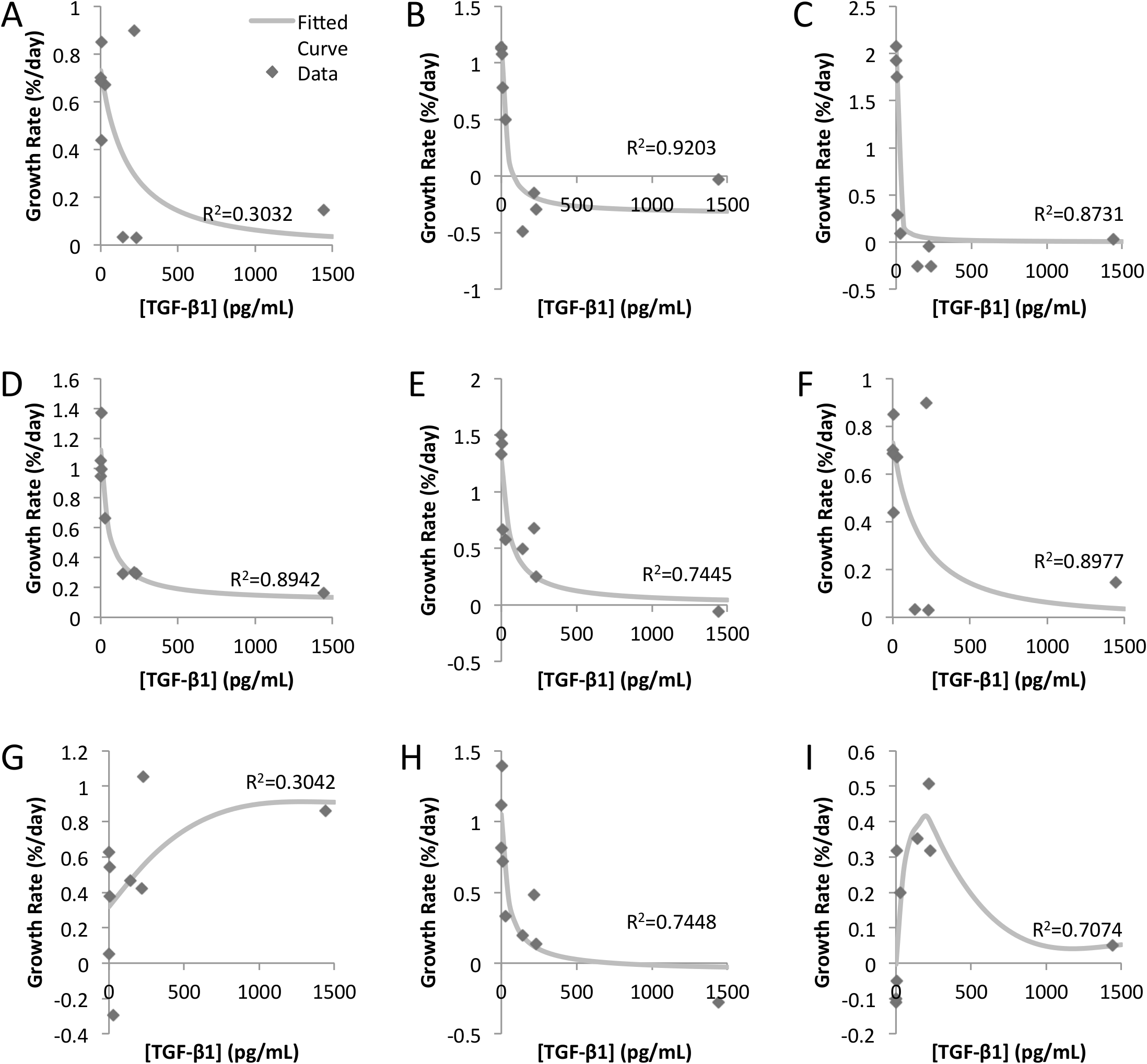

Supplement: S1 Fig — The correlations are used to calculate the growth rates at each time step of the model. Each relates the growth rate in % change per day to the current concentration of TGF-β1. [A] Group 1 [B] Group 2 [C] Group 3 [D] Group 4, [E] Group 5 [F-I] Group 6, [F] NK [G] T [H] ERY [I] NEUT. (TIF) [file pone.0137392.s001.tif]

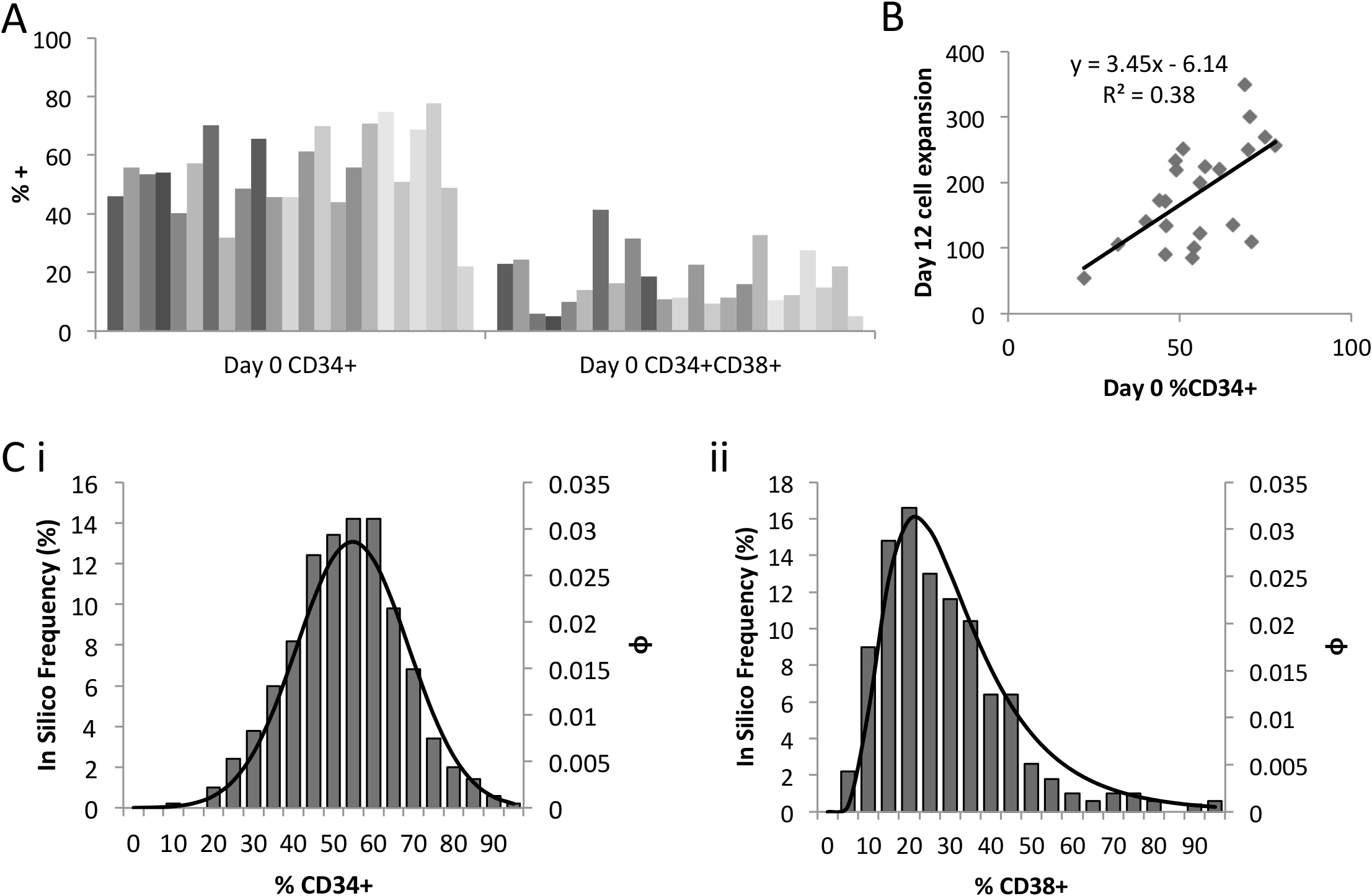

Supplement: S2 Fig — [A] The input cell population is highly variable, even after Lin- or CD34+ cell selection. [B] This variability has an effect on cell expansion, as %CD34+ starting cells is positively correlated with cell expansion (adapted from Csaszar et al. 2014 [20]). [C] The model incorporates two distributions for (i) CD34+ and (ii) CD38+ (as % of CD34+) to capture this variability. The primary y-axis corresponds to the observed frequency (n = 500 in silico replicates) while ϕ is the underlying probability density function used to generate the distributions. (TIF) [file pone.0137392.s002.tif]

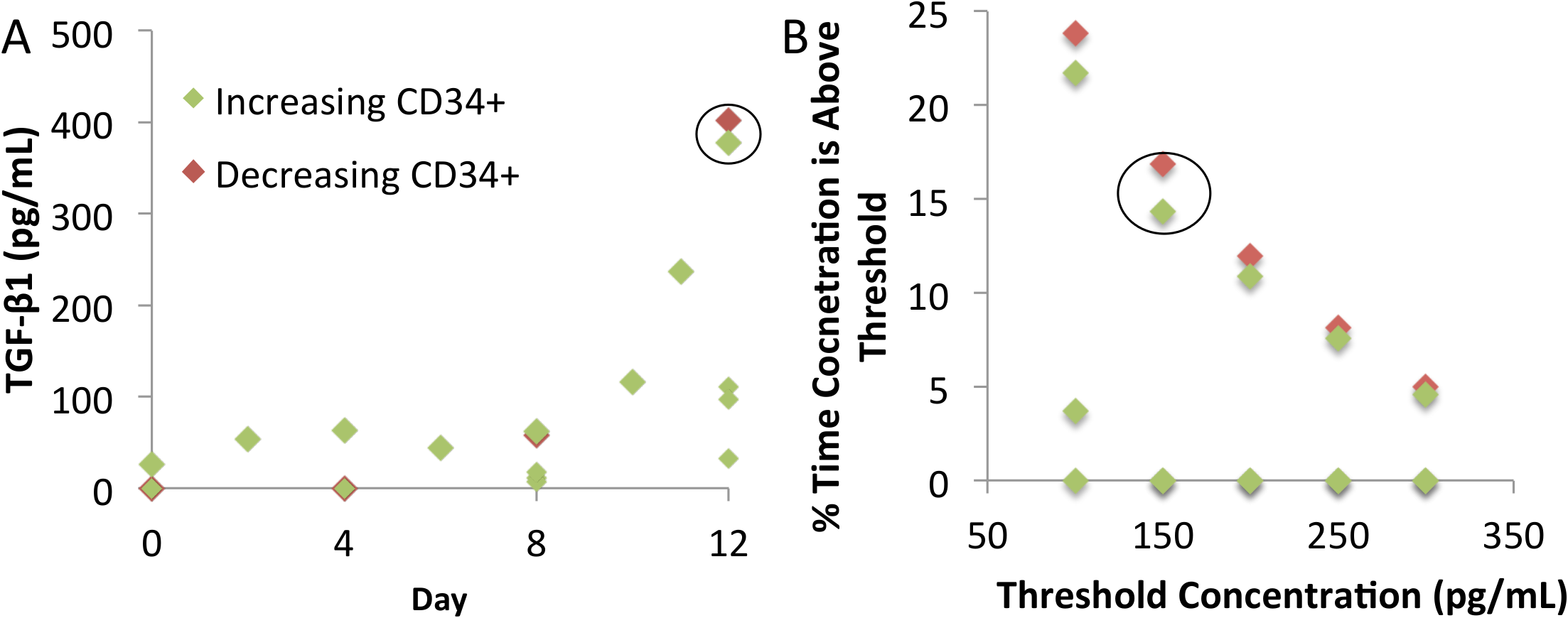

Supplement: S3 Fig — Conditions with a decrease in CD34+ cell number in the last days of culture can be differentiated from those with continued expansion [A] Concentration of TGF-β1 was predictive of differentiation at the end of culture, with a threshold of 400 pg/mL. [B] Cumulative culture times at high factor concentrations were predictive of differentiation. The threshold was set at 15% above 150 pg/mL. (TIF) [file pone.0137392.s003.tif]

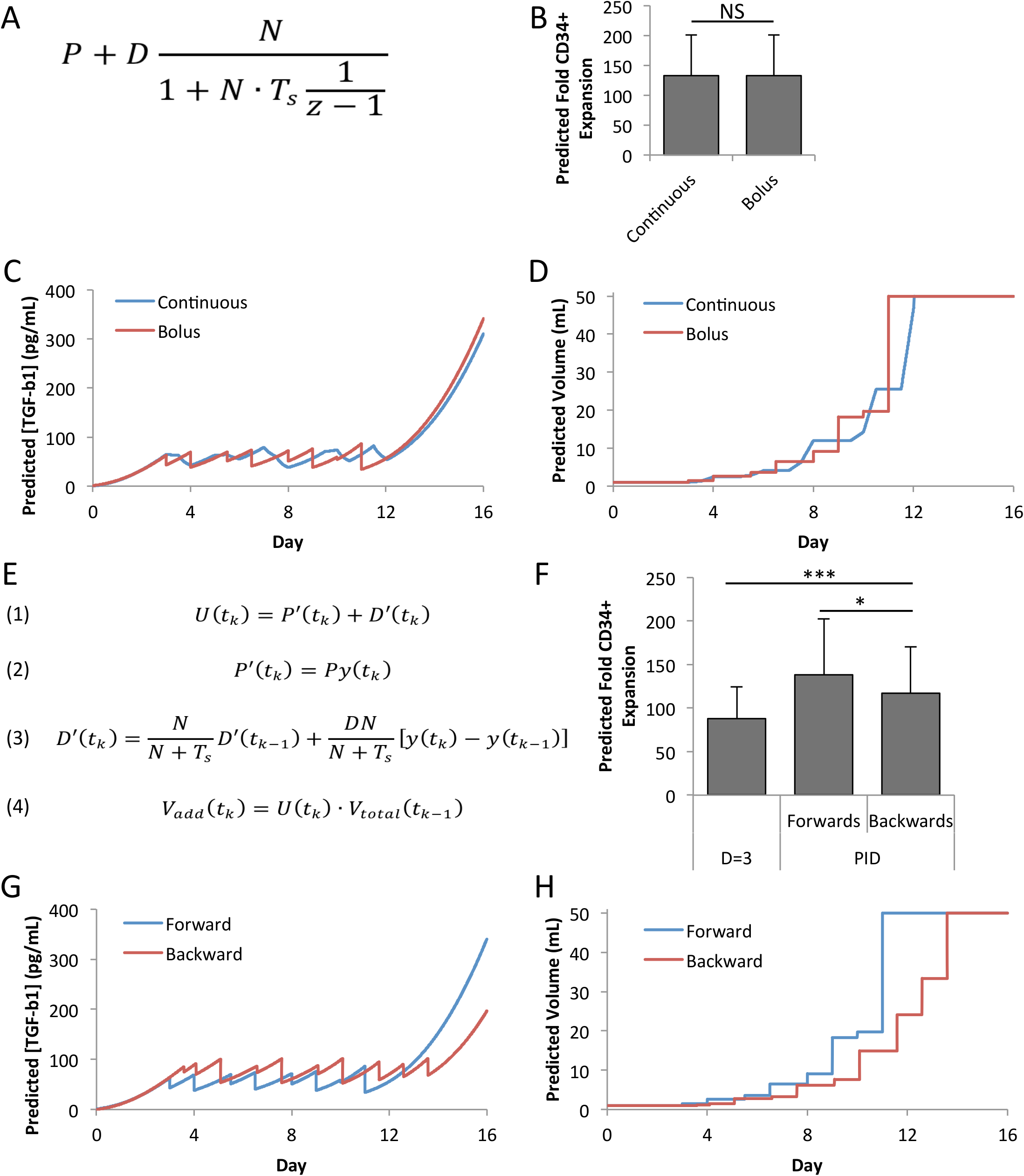

Supplement: S4 Fig — [A] The PID controller was designed to determine a media flow rate using a forward Euler approximation of the derivative. [B] When the controller output is calculated as a bolus delivery of media, as during manual implementation of the controller, no change is predicted in CD34+ expansion (n = 100). Predicted [C] concentration and [D] culture volume trajectories for the previously mentioned representative sample highlight the differences between the media delivery methods. Manual implementation of the controller requires the use of a backwards approximation of the derivative. [E] The controller output is calculated at each time step k using equations 1–3. If U(t k )>0, the media bolus is calculated using equation 4. [F] This calculation method lowers the predicted CD34+ cell expansion, but it remains an improvement over the linear dilution schemes (n = 100). Predicted [G] concentration and [H] volume trajectories for the representative sample highlight that backwards differentiation results in an extended controller action phase but higher average factor concentration. * p<0.05, *** p<0.001. (TIF) [file pone.0137392.s004.tif]

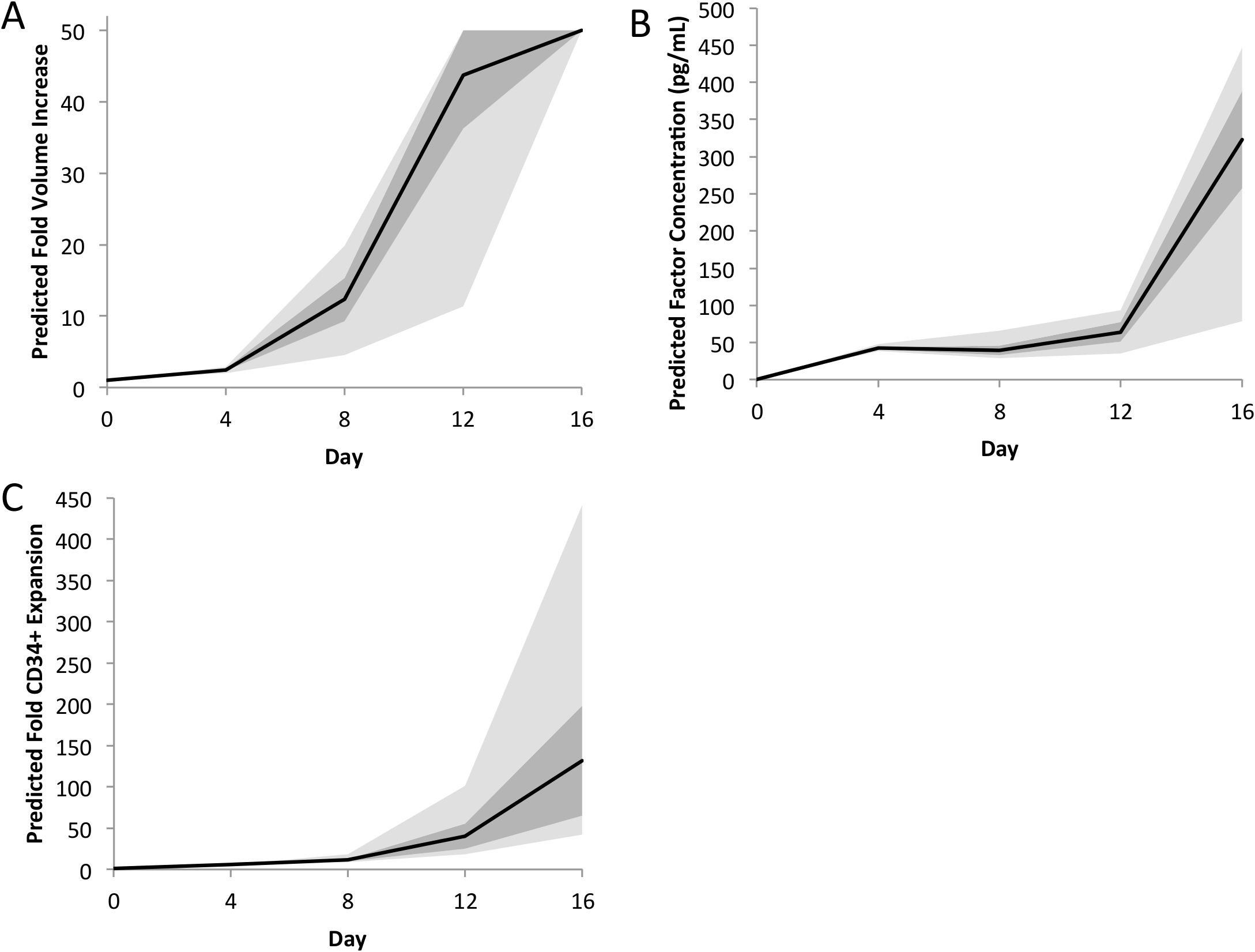

Supplement: S5 Fig — [A] The model predicts a broad range of possible volume trajectories when implemented with the PID controller. The solid black line represents the average value of 100 in silico replicates. The dark grey band shows the mean ± 1 standard deviation. The light grey band shows the full range of predicted values. [B] The model correspondingly predicts a broad range of possible TGF-β1 concentrations. [C] This results in a wide range of predicted CD34+ expansion, representative of population-level biological variability between units of cord blood. (TIF) [file pone.0137392.s005.tif]

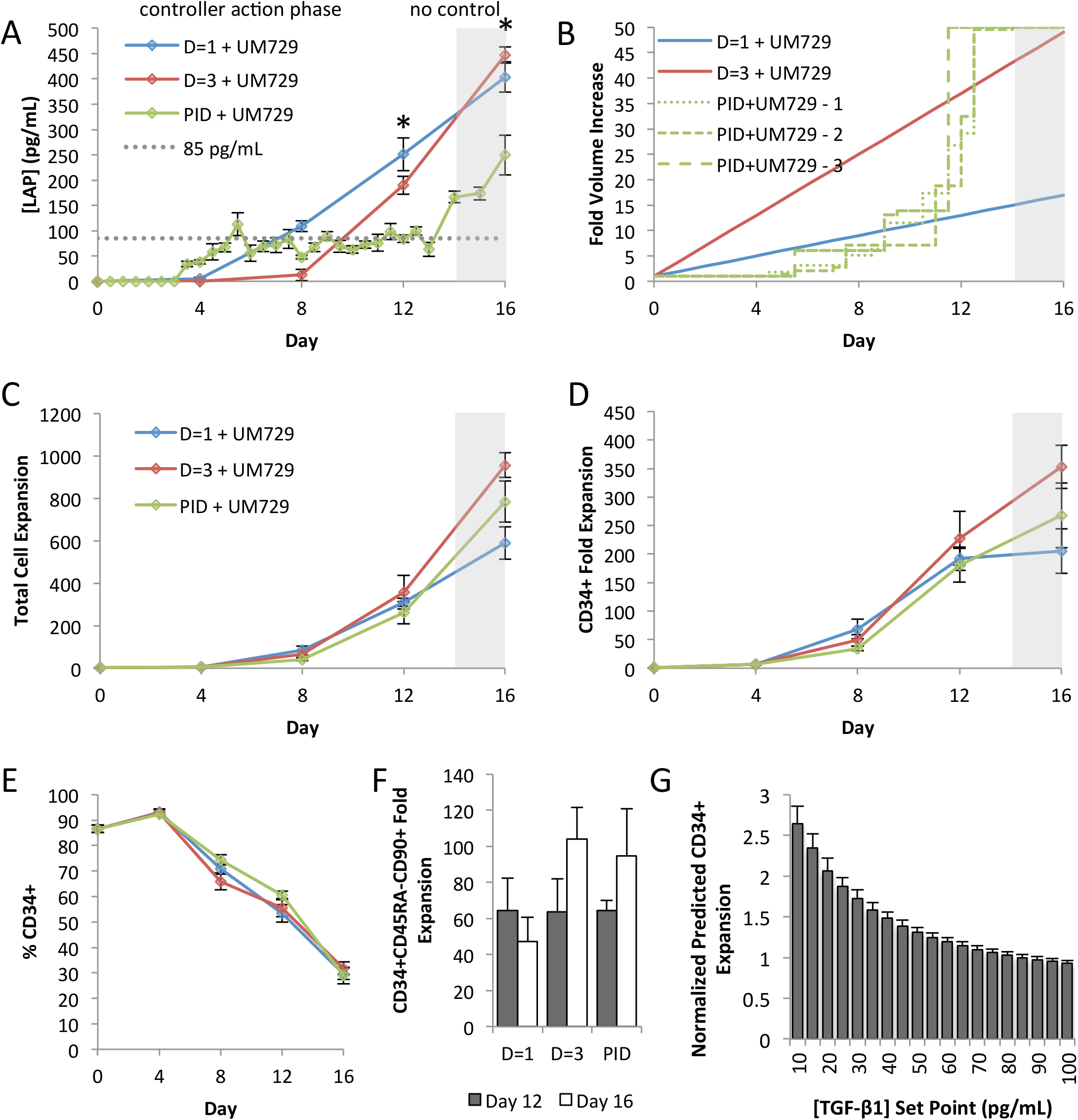

Supplement: S6 Fig — [A] Average LAP concentration time course demonstrates that the PID controller maintains a lower factor concentration (n = 3) than linear medium dilution strategies during the controller action phase, though the magnitude of this difference is small compared to 3-factor conditions. [B] Volume trajectories for D = 1, D = 3 and 3 PID controlled samples supplemented with UM729 shows controller action is extended to day 12. [C] Total cell expansion compared between dilution strategies. PID control with UM729 is not significantly different from controls at day 12 or day 16 (n = 3). [D] CD34+ cell expansion compared between dilution strategies. Again, PID control with UM729 is not significantly different from controls at day 12 or day 16 (n = 3). [E] Surface marker analysis of CD34+ frequency during culture that PID control has no effect when combined with UM729 (n = 3). [F] PID control with UM729 does not offer any advantages for expansion of the HSC-enriched population, CD34+CD45RA-CD90+ (n = 3). [G] In silico modeling suggests that PID control would have synergistic effects with UM729 when implemented with a lower set point. * p<0.05. (TIF) [file pone.0137392.s006.tif]

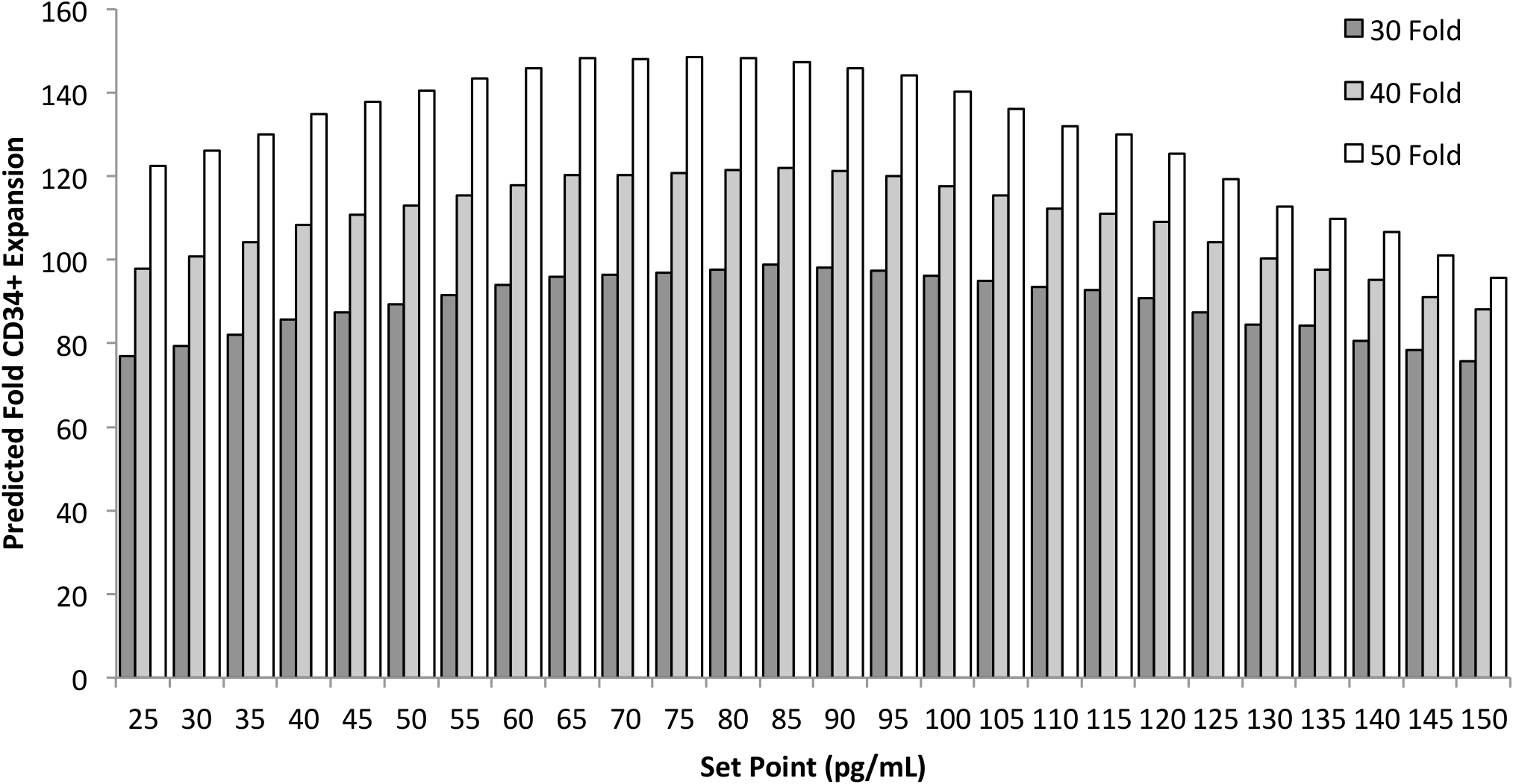

Supplement: S7 Fig — The model predicts a TGF-β1 set point of 85 pg/mL maximizes CD34+ cell expansion at fold volume increases of 30, 40 and 50 (n = 100). (TIF) [file pone.0137392.s007.tif]
