# Supplementary material for: Proportional-Integral-Derivative (PID) Control of Secreted Factors for Blood Stem Cell Culture
Source: PLoS One. 2015 Sep 8;10(9):e0137392. doi: 10.1371/journal.pone.0137392 (PMC4562642; doi:10.1371/journal.pone.0137392)
Supplement: S1 Table — (DOCX) [file pone.0137392.s008.docx]

**S1 Table. Phenotype definitions used for model development.**

| **Cell Type** | **Surface Marker Definition** |
| --- | --- |
| Hematopoietic Stem Cell (HSC) | CD34^+^CD38^-^CD45RA^-^CD49f^+^ |
| Multipotent Progenitor (MPP) | CD34^+^CD38^-^CD45RA^-^CD49f^-^CD90^-^ |
| Multilymphoid Progenitor (MLP) | CD34^+^CD38^-^CD45RA^+^CD90^-^CD10^+^ |
| Common Myeloid Progenitor (CMP) | CD34^+^CD38^+^CD45RA^-^CD135^+^CD7^-^CD10^-^ |
| Granulocyte and Monocyte Progenitor (GMP) | CD34^+^CD38^+^CD45RA^+^CD135^+^CD7^-^CD10^-^ |
| Megakaryotic and Erythroid Progenitor (MEP) | CD34^+^CD38^+^CD45RA^-^CD135^-^CD7^-^CD10^-^ |
| Natural Killer (NK) | CD34^-^CD7^+^CD3^-^ |
| T Cell (T) | CD34^-^CD7^+^CD3^+^ |
| B Cell (B) | CD34^-^CD19^+^ |
| Megakaryocyte (MK) | CD34^-^CD41^+^ |
| Erythrocyte (ERY) | CD34^-^CD235a^+^ (CD71^-/+^) |
| Neutrophil (NEUT) | CD34-CD11b^+^CD16^+^ |
| Eosinophil and Basophil (EOS/BASO) | CD34^-^CD11b^-^CD16^-^CD33^+^CD14^+^ |
| Monocyte and Dendritic Cell (MONO/DC) | CD34^-^CD11b^-^CD16^-^CD33^+^CD14^-^ |
