# Supplementary material for: Proportional-Integral-Derivative (PID) Control of Secreted Factors for Blood Stem Cell Culture
Source: PLoS One. 2015 Sep 8;10(9):e0137392. doi: 10.1371/journal.pone.0137392 (PMC4562642; doi:10.1371/journal.pone.0137392)
Supplement: S2 Table — (DOCX) [file pone.0137392.s009.docx]

**S2 Table. Phenotype Groups.**

| **Group** | **Phenotypes** | **Gene Expression Value** |
| --- | --- | --- |
| 1 | GMP | 0.24990 |
| 2 | MPP, MEP | 0.38690 |
| 3 | HSC, MLP, CMP | 0.34846 |
| 4 | MK, EOS/BASO | 0.93285 |
| 5 | MONO/DC, B | 0.79107 |
| 6 | NK, T, ERY, NEUT | 0.81588 |
